# Examining the impact of the early stages of the COVID-19 pandemic period on youth cannabis use: adjusted annual changes between the pre-COVID and initial COVID-lockdown waves of the COMPASS study

**DOI:** 10.1186/s12889-021-11241-6

**Published:** 2021-06-21

**Authors:** Scott T. Leatherdale, Richard E. Bélanger, Rabi Joël Gansaonré, Karen A. Patte, Margaret deGroh, Ying Jiang, Slim Haddad

**Affiliations:** 1 School of Public Health Sciences, University of Waterloo, 200 University Avenue, Waterloo, ON N2L 3G1 Canada; 2 VITAM - Centre de recherche en santé durable, Université Laval, Quebec, Canada; 3 Department of Pediatrics, Faculty of Medicine, Université Laval, Quebec, Canada; 4 Department of Health Sciences, Brock University, St. Catharines, Canada; 5 Applied Research Division, Public Health Agency of Canada, Ottawa, Canada; 6 Department of Social and Preventive Medicine, Faculty of Medicine, Université Laval, Quebec, Canada

**Keywords:** COVID-19, Pandemic, Youth, Cannabis, Adolescents, Prospective, Cohort

## Abstract

**Background:**

Given the high rates of cannabis use among Canadian youth and that adolescence is a critical period for cannabis use trajectories, the purpose of this paper was to examine the effect of the early stages of the COVID-19 pandemic period on youth cannabis use in the context of a natural experiment.

We used 3-year linked data from the COMPASS study, including 7653 Canadian (Quebec, Ontario) adolescents from which 1937 completed all 3 survey waves (pre-COVID-19 [2018, 2019] and online [2020] during the early pandemic period [May–July 2020]). Structural equation modeling (SEM) and double difference (DD) models were used to estimate pre-COVID-19 to initial COVID-19 pandemic period change (2019–2020) in cannabis use (monthly, weekly, daily) compared to 2018 to 2019 change to adjust for age-related effects. Models were adjusted for age of entry into the cohort and sociodemographic characteristics.

**Results:**

In the SEM and DD models, monthly, weekly, and daily cannabis use increased across all waves; however, the expected increases from the pre-COVID-19 wave (2019) to the initial COVID-19 period wave (2020) were lesser relative to the changes seen across the 2018 to 2019 waves. The cross-sectional data from May to July 2020 identified that the majority of youth who use cannabis did not report increased cannabis use due to COVID-19 or using cannabis to cope with COVID-19.

**Conclusion:**

During the early stages of the COVID-19 pandemic period, there does not appear to be a detrimental effect on youth cannabis use, when adjusted for age-related changes. Further prospective research is needed to explore the impact of the ongoing pandemic response on youth cannabis use onset and progression.

## Background

The rate of cannabis use among youth is higher in Canada than most other countries globally [1, 2]. In 2015, just over 1 in 4 Canadian youth between 15 and 19 years of age reported consuming cannabis in the past year [1]; making cannabis the most widely used illicit drug among young Canadians. More recent data suggest similar findings. In a large cross-sectional sample of Ontario youth (grades 9 to 12) in 2019, 22% of respondents reported using cannabis products in the past year, with over 40% of grade 12 respondents reporting cannabis use [3]. There is also longitudinal evidence that patterns of cannabis use among youth can rapidly change, with the escalation of use more common than reduction or cessation over a two-year follow-up across secondary school [4, 5]. Since cannabis use habits are commonly established in adolescence and can profoundly affect subsequent trajectories as youth mature and progress into adulthood [6, 7], improving our understanding of cannabis use and factors associated with changes in cannabis use behaviour over time is critical for informing future prevention efforts. This is especially important given the many adverse outcomes associated with early onset and frequent cannabis use [7–10].

On March 11, 2020, the World Health Organization announced that COVID-19 was a global pandemic [11]. The onset of the COVID-19 pandemic resulted in the federal and provincial governments in Canada enacting emergency lockdowns and behavioural precautions in the first few months of the pandemic (March to July 2020) that have directly affected youth given the immediate and unprecedented disruption to their traditional routines. Restrictions during the early pandemic lockdown included closures to in-person learning in schools and transitioning to online learning platforms, the cancellation of recreational sports and facilities for physical activities, the closure of leisure-based facilities (e.g., malls, movie theatres), and social restrictions that limited the ability to socialize with friends/peers and increased home-based confinement with parents/guardians. While it has been proposed that the lack of structure from in-person schooling and social activities will likely have a lasting impact on youth into the future [12], prospective evidence of the impact of these restrictions on youth from the pre-pandemic to early pandemic period are lacking. Emerging evidence suggests that during the early pandemic period, substance use (including cannabis) increased among Canadian adults [13], but the impact on youth cannabis use remains unclear. Moreover, no prospective studies with pre-pandemic data have examined the impact of COVID-19 during the early pandemic period on cannabis use in adolescents.

For the vast majority of Canadian youth, the COVID-19 restrictions that occurred during the early pandemic period would be an unprecedented experience. It seems inevitable that these restrictions would likely have had some sort of effect (positive or negative) on cannabis use in the short term [14]. For instance, according to the Canadian Centre on Substance Use and Addiction (CCSA), conditions surrounding COVID-19 (e.g., stress and anxiety, social isolation due to physical distancing, closing of non-essential facilities) are likely to lead to increased cannabis use [13], with early evidence suggesting that some youth used cannabis as a coping-related behaviour to regulate affect during the early pandemic period [15]. Conversely, given that cannabis use in this age group can often been seen as opportunistic with peers as a means for enhancing social experiences [16], and that most youth consume substances for social reasons and may be less likely to do so alone [17], it is also possible that when social interaction was limited during the early pandemic period, cannabis use may have actually declined. This is supported with evidence from a small cross-sectional sample of Ontario youth demonstrating that the prevalence of cannabis use decreased 3 weeks after social distancing measures came into effect [15]. Given the ambiguity in the limited available evidence, the purpose of this study is to leverage pre- and early-pandemic data from an ongoing Canadian prospective cohort study of youth in two Canadian provinces (Ontario and Quebec) to evaluate the effect of COVID-19 during the early stages of the pandemic period on youth cannabis use as a natural experiment. Specifically, we tested the hypothesis that the expected escalation trajectory for cannabis use among youth in our sample would decrease between the pre-COVID-19 period and the early initial pandemic period (May–July 2020), to a greater extent than expected with age. In addition, using cross-sectional data collected during the early pandemic period, we also examine solitary use of cannabis, changes in cannabis use as a result of COVID-19, and using cannabis to cope with COVID-19 related negative effects among past year cannabis users.

## Methods

The Cannabis use, Obesity, Mental health, Physical activity, Alcohol use, Smoking, and Sedentary behaviour (COMPASS) Study is an ongoing prospective study designed to collect hierarchical health data once annually from a rolling cohort of students in grades 9 through 12 (Secondary I-V in Quebec) and the secondary schools they attend [18, 19]. The student-level data are collected annually during the school year (e.g., Sept. 2017 to May 2018, referred to as the 2018 year) via a self-reported questionnaire across multiple content domains (including cannabis use), using an active information passive-consent protocol [the parent(s)/guardian(s) of each eligible student are informed of all study protocols and provided the opportunity to withdraw their child from participating [20]]; students could decline to participate at any time [20]. All procedures were approved by the University of Waterloo Office of Research Ethics (reference number 30118), CIUSSS de la Capitale-Nationale–Université Laval (#MP-13-2017-1264), and appropriate school board committees. A full description of the COMPASS study methods are available online [19].

### Design

To evaluate the effect of COVID-19 as a natural experiment, we used linked longitudinal COMPASS data collected from students that attended a convenience sample of 43 schools in Ontario (*N* = 20) and Quebec (*N* = 23) that participated in the both the Wave 6 (2018; 81.8% response rate) and Wave 7 (2019; 84.2% response rate) in-person paper-based survey, and the Wave 8 (2020; 29.2% response rate) online survey. In the 2018 and 2019 waves, all student-level data in these 43 schools were collected using a paper-based survey in class time [the in-person paper-based data collection procedures are described in detail elsewhere [21]]. In 2020, these 43 schools were closed for in-person learning due to COVID-19 social distancing restrictions, so all of the student-level data in these schools were collected using an online Qualtrics® survey completed at home between May 01 and July 06, 2020 [the online data collection procedures are described in detail elsewhere [22]]. Longitudinal student data are obtained through an anonymous linking process which allows matching of student responses over time using a self-generated identification code created from five measures asked at the beginning of the questionnaire where the responses to those questions should not change over time (e.g., second letter of your first full name, the month you were born) [23]. These five measures, combined with a student’s sex, are used to create a six-digit school specific identifier for each participant that can be linked across waves; this unique identifier does not involve recording the names of participants hence maintaining student confidentiality [a detailed technical report is available that describes the specific data linkage measures and linkage algorithms [23]]. To examine how the students reported that COVID-19 effected their cannabis use, we used cross-sectional data from all students who participated in the Wave 8 (2020) online data collection from the 43 schools.

### Participants

Within these 43 schools, data from 7653 students (grades 9 and 10 in Ontario or Secondary I-II in Quebec) in the 2018 data collection wave were successfully linked to the 2019 data collection wave, of which 2099 were then successfully linked to the 2020 online data collection wave. Among the students successfully linked between 2018 and 2019, 7567 (98.9%) in 2018 and 7548 (98.6%) in 2019 provided data on their cannabis use. Among the students then successfully linked to the 2020 online wave, 1937 (92.2%) provided data on their cannabis use. Participants with missing cannabis use data in 2018, 2019 or 2020 were excluded from our models using the linked longitudinal sample. Cross-sectional student-level data were available from 7496 students (grades 9 to 12 in Ontario and Secondary I-V in Quebec) who participated in the 2020 online data collection in the 43 schools.

### Measures

Consistent with national surveillance measures on youth cannabis use [24], each year students were asked, “In the last 12 months, how often did you use marijuana or cannabis?” and responded with one of 9 options: “I have never used marijuana”, “I have used marijuana but not in the last 12 months”, “Less than once a month”, “Once a month”, “2 or 3 times a month”, “Once a week”, “2 or 3 times a week”, “4 to 6 times a week”, and “Every day”. Consistent with previous research [4], responses were recoded into “*monthly use”* if reported use was once to 3 times a month, “*weekly use”* if use ranged from once to 6 times a week, and “*daily use*” if use was reported as everyday. In the 2020 online survey, students were also asked “in the last 30 days, how many times have you used marijuana or cannabis when you were all by yourself” s (never, once, twice, 3 or more times, I don’t know), “How has your life changed because of COVID-19? My cannabis use has … ” (increased, stayed the same/not applicable, decreased), “How have you been coping with changes related to COVID-19? Mark all that apply (20 options listed)” (Using cannabis/marijuana). Covariates included sex (female, male), age in 2018 [13–15], weekly spending money (≤$5, $6–$10, $11–$20, ≥$20), school connectedness (5 to 25), and province (Ontario, Quebec).

### Analyses

Longitudinal-linked student-level data from the 43 schools were used to examine the adjusted annual changes in cannabis use (monthly, weekly, and daily) among students in the pre-COVID waves (2018 and 2019) and during the waves straddling the early pandemic period (2019 and 2020). Given the nature of the COVID-19 pandemic, it is not possible to have control group data (i.e., a group of students not exposed to COVID-19 restrictions but still provided 2020 data). As such, the hypothesis of a different progression of annual prevalence of cannabis use as a function of COVID-19 in each of the two intervals was tested using double-difference (DD) models, where the first difference consists of subtracting the mean potential outcomes of a student in 2018 and 2019 and the second difference compares the same student’s responses in 2019 and 2020 [25]. The DD provides an estimation of the average effect of the early stages of the COVID-19 pandemic response on student cannabis use.

Mean potential outcomes and average treatment effects were obtained using Stata 15 Generalized Structural Equation Modeling (GSEM) routines (Stata Corp, College Station, TX). To control for unobserved heterogeneity, we used a fixed effect method using GSEM as recommended by Allison [26, 27], given the advantages of simultaneously controlling for time-invariant unmeasured confounders and producing final estimates for time-invariant predictors (sex and age at entry into the cohort). A structural model was developed for each of the three outcomes; each model included three equations (one for each year). Following Allison’s approach [26], each set of equations included a vector of invariant predictors (sex and age at entry into the cohort) as well as a latent term (Alpha) representing all other unobserved stable differences between individuals. Full Information Maximum Likelihood method was used to adjust for missing covariate data. Robust estimators accounted for school clustering.

An additional complication resides in the self-selection process encountered in the Wave 8 (2020) online survey due to the lower response rate in 2020 and subsequent bias. To account for self-selection in 2020, the initial models (Type 1 Model) were supplemented by Heckman-type sample selection models (Models 2 and 3) [28]. A selection equation was first estimated (probit equation) using a set of predictors of self-selection (age, sex, weekly spending money, school-connectedness, province). Then, the inverse Mills Ratio was generated and introduced as an additional explanatory variable into the 2020 equation of each SEM model to correct for the selection bias.

The DD calculations for each outcome were performed in three steps, aimed respectively at obtaining the mean predicted values under the counterfactual scenarios, computing simple differences, and estimating the causal effect through DD. The 95% confidence intervals were estimated based on robust Huber-White standard errors. The structural equation models (SEM) were used to control for time-invariant confounders and apply a sample selection correction to control for self-selection bias. Considering that few youth who are regular users of cannabis spontaneously reduce their use [4], changes observed in this DD approach are likely a result of the early stages of the COVID-19 pandemic period having an effect. We assume that without such impact, the annual change (*expected increase*) in cannabis use among students would be the same between the pre-COVID-19 period (2018 to 2019) and the initial COVID-19 period (2019 to 2020).

Using the cross-sectional student-level data collected from 7496 students attending these 43 schools in 2020, we examined solitary use of cannabis, changes in cannabis use as a result of COVID-19, and using cannabis to cope with COVID-19 related negative affect among students who reported past year cannabis use.

## Results

Within our three-year linked longitudinal sample of 1937 students, the mean age in 2018 was 14.1 (±1.0) years, just over half (53.1%) identifying themselves as females, and 53.5% attended a school in Quebec. At baseline in 2018, 5.7% (±0.2) of students reported monthly use of cannabis, 2.4% (±0.2) reported weekly use of cannabis, and 0.6% (±0.1) reported daily use of cannabis.

Refer to Table 1 for observed rate for cannabis use among eligible secondary school students across the three waves. Accordingly, between 2018 and 2019 there was an increase in the prevalence of cannabis use in the sample, and then a decrease between 2019 and 2020, likely as a function of bias introduced by the online survey and the subsequent decrease in sample size. As such, for interpretation of our models, the focus is on the adjusted means derived from the GSEM models for each outcome. When examining the adjusted estimates, across all three outcomes modelled, there was an increase in cannabis use between each wave, with the highest rates of cannabis use during the last wave (2020). As shown, for each outcome, the average discrete change between years decreased between the pre-COVID-19 and early stages of the COVID-19 pandemic response. Across all three cannabis use outcomes modelled, even after accounting for predictors of self-selection in the 2020 sample, the negative estimated causal effects shown for the difference-in-difference results (− 5.7% for monthly use, − 3.0% for weekly use, and − 0.3% for daily use), support the hypothesis that there was a reduction in the expected escalation of cannabis use within the sample during the early stages of the COVID-19 pandemic period. The reduction in the expected escalation of cannabis use was the largest among those reporting less frequent use during pre-COVID cycles.

**Table 1:** Observed and adjusted cannabis use means, discrete change of cannabis use indicators over survey waves, and estimated causal effect of COVID-19 on cannabis use by difference-in-difference among eligible students attending the 43 linked-longitudinal COMPASS schools across the three study waves (2018, 2019, 2020)

			Cannabis Use					
			***Monthly Use***		***Weekly Use***		***Daily Use***	
	Wave	N	Observed Mean (SD)	Adjusted Estimate^a^ Mean (95% CI)	Observed Mean (SD)	Adjusted Estimate^a^ Mean (95% CI)	Observed Mean (SD)	Adjusted Estimate^a^ Mean (95% CI)
**Observed and Adjusted Outcome Mean**	2018	7567	5.7 (0.2)	5.7 (4.9, 6.5)	2.4 (0.2)	2.4 (1.8, 3.0)	0.6 (0.1)	0.6 (0.3, 0.9)
	2019	7548	12.1 (0.3)	12.0 (11.0, 12.9)	6.1 (0.2)	6.4 (5.2, 7.6)	1.5 (0.1)	1.4 (1.1, 1.7)
	2020	1937	7.5 (0.3)	12.6 (10.7, 14.4)	3.9 (0.2)	7.4 (5.7, 9.2)	0.8 (0.1)	1.9 (1.4, 2.5)
			**Difference Mean (95% CI)**		**Difference Mean (95% CI)**		**Difference Mean (95% CI)**	
**Average Discrete Change**^b^	2019–2018 (pre-COVID-19 period)		6.3 (5.2, 7.4)		4.0 (2.5, 5.4)		0.8 (0.4, 1.2)	
	2020–2019 (early COVID-19 period)		0.6 (−1.2, 2.3)		1.0 (− 1.5, 3.5)		0.5 (− 0.1, 1.0)	
			**Difference-in-Difference** **(95% CI)**		**Difference-in-Difference** **(95% CI)**		**Difference-in-Difference** **(95% CI)**	
**Estimated Causal Effect**^b^	(2020-2019) - (2019-2018)		− 5.7 (− 8.0, − 3.4)		−3.0 (− 6.7, 0.8)		− 0.3 (− 1.1, 0.4)	

Notes: 95% CI (confidence interval)

^a^ Fixed effect model with a lagged variable as the outcome, controlling for time-invariant confounders but constraining sex and age effects on the outcome to be fixed across time, and sample selection correction with the predictors of age, sex, weekly spending money, school connectedness, and province.

^b^ based on adjusted estimates.

**Fig. 1:**
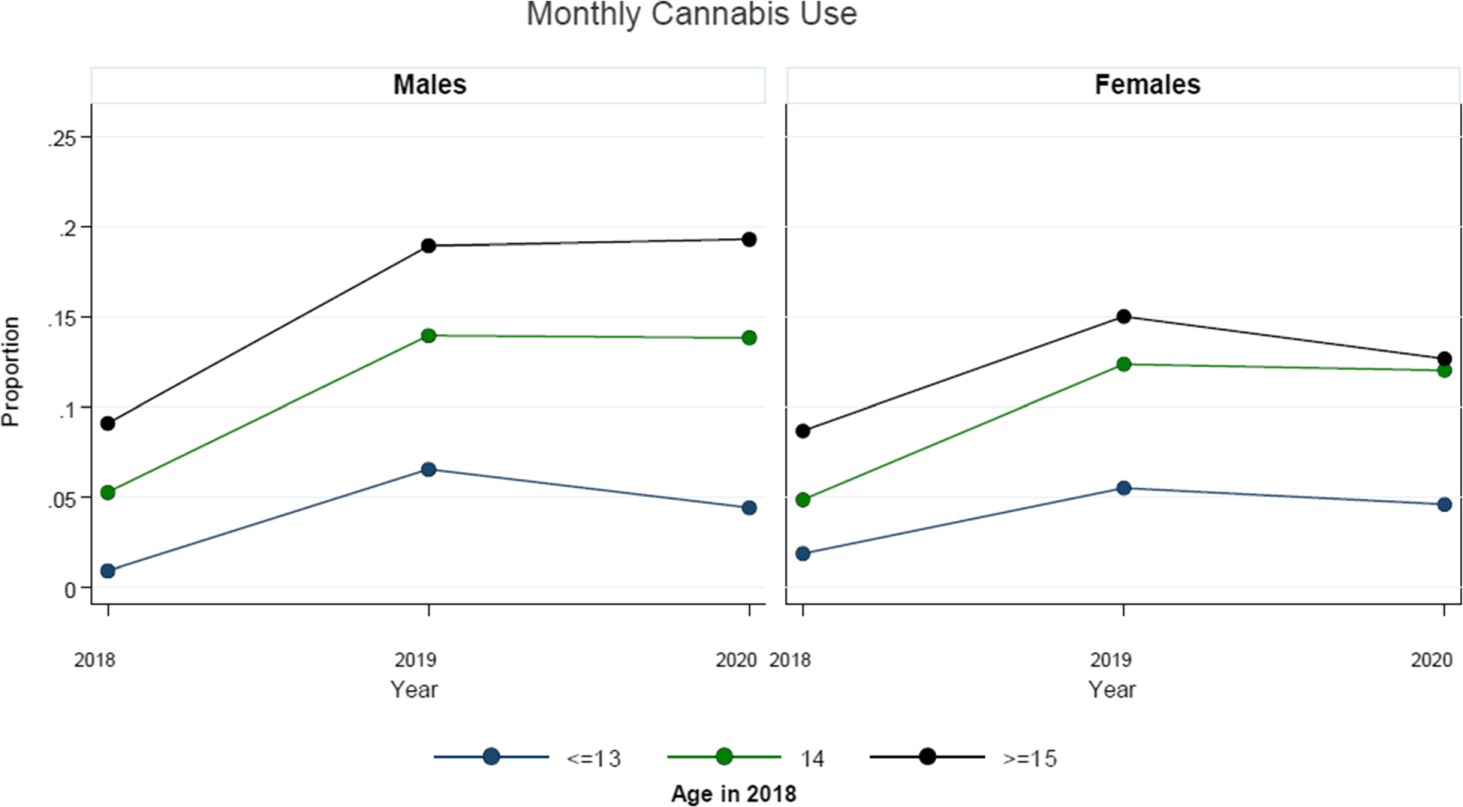
Average adjusted predictions of monthly cannabis use over survey waves, stratified by sex and age (at entry into cohort in 2018), among eligible students attending the 43 linked-longitudinal COMPASS schools across the three study waves (2018, 2019, 2020)

**Fig. 2:**
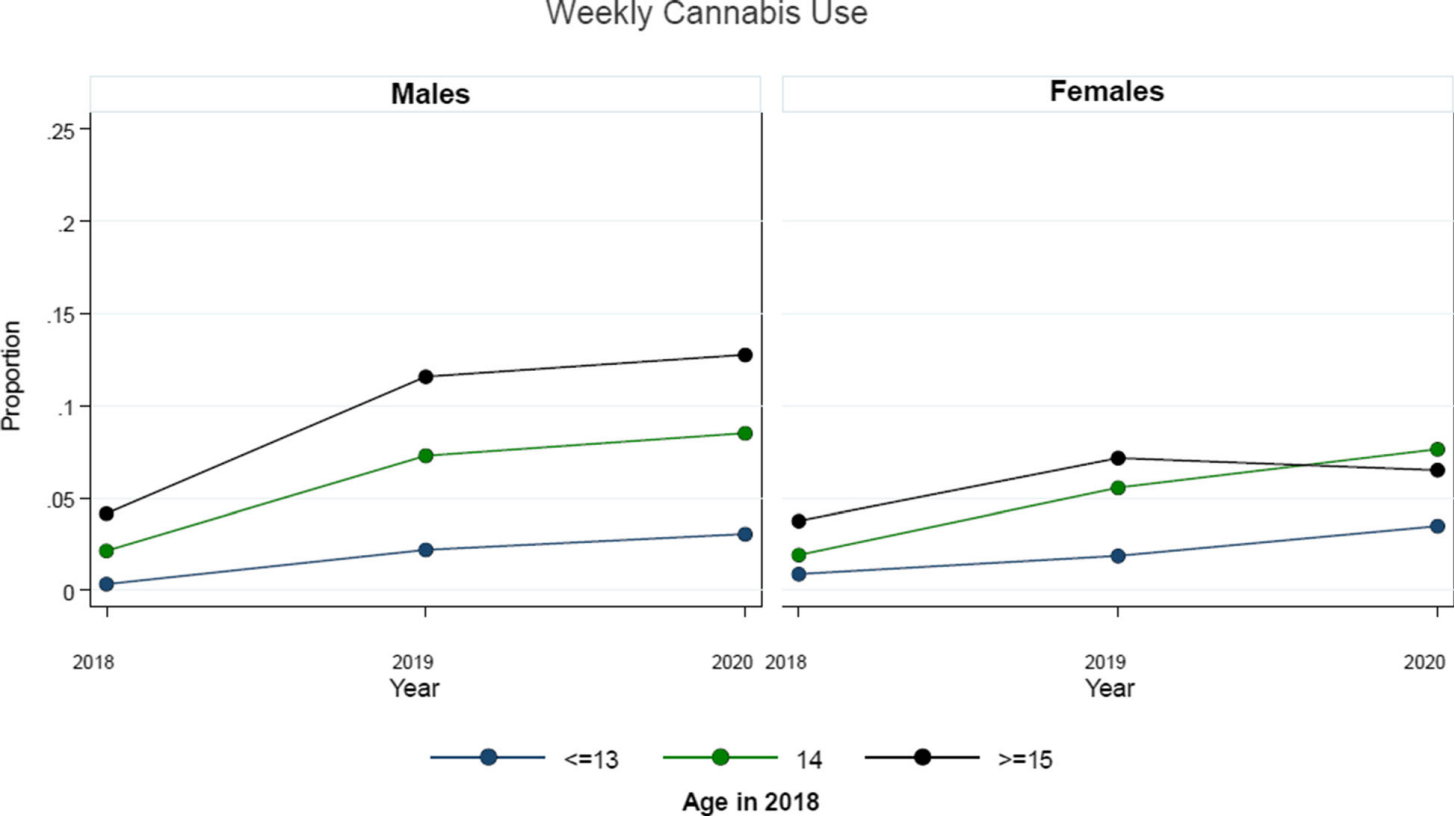
Average adjusted predictions of weekly cannabis use over survey waves, stratified by sex and age (at entry into cohort in 2018), among eligible students attending the 43 linked-longitudinal COMPASS schools across the three study waves (2018, 2019, 2020)

**Fig. 3:**
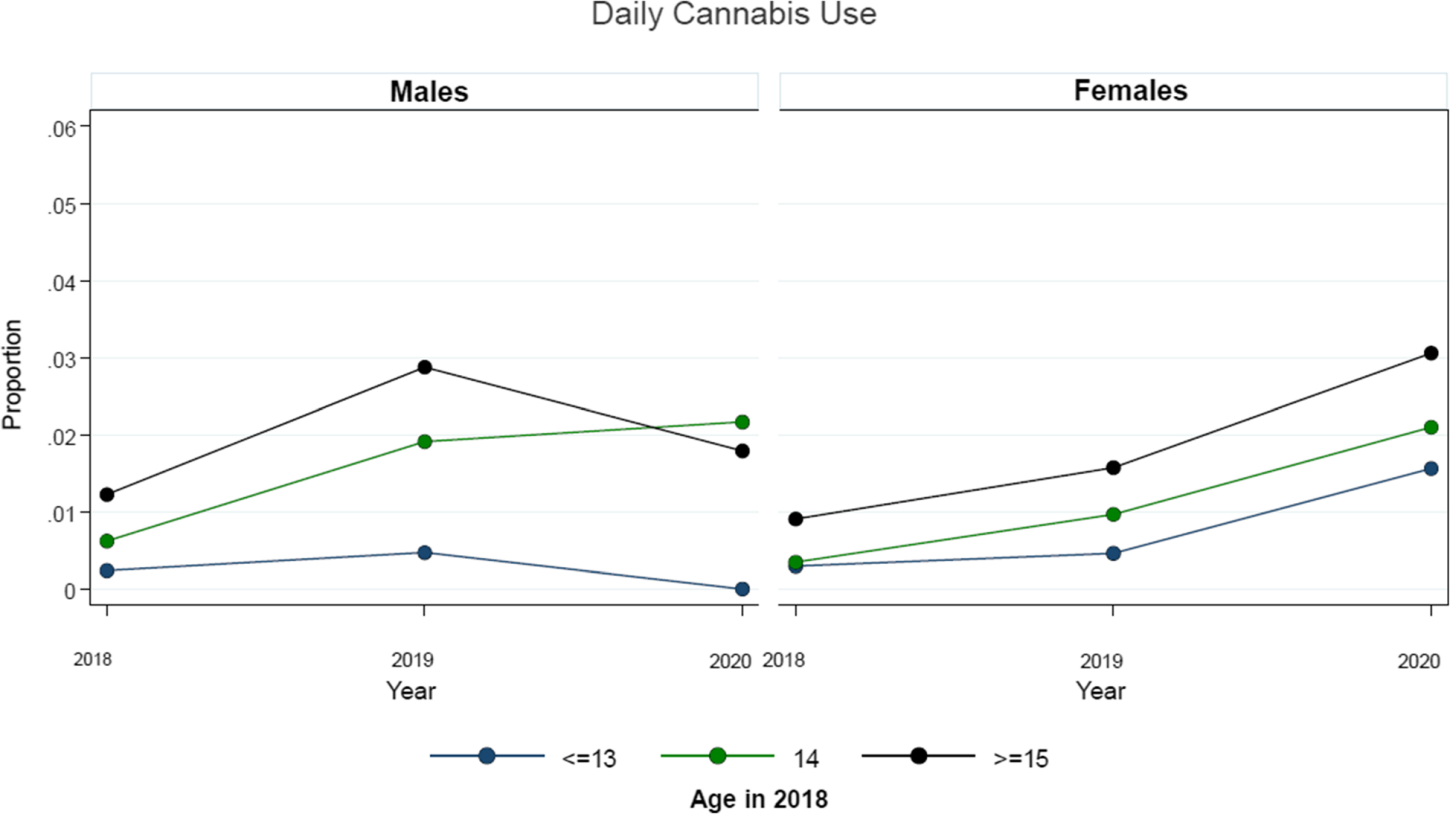
Average adjusted predictions of daily cannabis use over survey waves, stratified by sex and age (at entry into cohort in 2018), among eligible students attending the 43 linked-longitudinal COMPASS schools across the three study waves (2018, 2019, 2020)

Table 2 presents the examination of the possible differential impact of the early stages of the COVID-19 pandemic response on cannabis use stratified by sex. As shown, it appears that there was a greater reduction in the expected escalation of cannabis use among males relative to females within the sample during the initial COVID-19 period. Figures 1 , 2, and 3 present the examination of the possible differential impact of the early COVID-19 pandemic period on monthly (Fig. 1), weekly (Fig. 2), and daily (Fig. 3) cannabis use stratified by sex and age at entry into the cohort (2018). As shown, it appears that there was a greater reduction in the expected escalation of monthly (Fig. 1) and weekly (Fig. 2) cannabis use among older students relative to younger students for both males and females within the sample during the initial COVID-19 period. However, while there was a greater reduction in the expected escalation of daily cannabis use among older male students relative to younger male students as shown in Fig. 3, there does not appear to be a reduction in the escalation of daily cannabis use among females of any age group.

**Table 2:** Average discrete change of cannabis use indicators over survey waves, and estimated causal effect of COVID-19 on cannabis use by difference-in-difference, stratified by sex, among eligible students attending the 43 linked-longitudinal COMPASS schools across the three study waves (2018, 2019, 2020)

		Cannabis Use					
		***Monthly Use***^a^		***Weekly Use***^a^		***Daily Use***^a^	
		Difference Mean (95% CI)		Difference Mean (95% CI)		Difference Mean (95% CI)	
		Female	Male	Female	Male	Female	Male
**Average Discrete Change**^b^	2019–2018 (pre-COVID-19 period)	5.0 (0.4, 0.6)	7.9 (6.4,9.4)	2.9 (1.2, 4.5)	5.2 (3.7, 6.8)	0.5 (0.1, 1.0)	1.2 (0.5, 1.8)
	2020–2019 (early COVID-19 period)	0.2 (−0.3, 0.3)	1.1 (− 0.9, 3.0)	0.9 (−1.7, 3.5)	1.1 (− 1.9, 4.1)	1.3 (0.5, 2.0)	− 0.4 (−1.4, 0.5)
		**Difference-in-Difference** **(95% CI)**		**Difference-in-Difference** **(95% CI)**		**Difference-in-Difference** **(95% CI)**	
		Female	Male	Female	Male	Female	Male
**Estimated Causal Effect**^b^	(2020-2019) – (2019-2018)	−4.8 (−8.2, −1.4) *p = 0.006*	−6.8 (− 9.7, −3.9) *p < 0.001*	−2.0 (−5.9, 2.0) *p = 0.325*	−4.1 (− 8.2, −0.1) *p = 0.045*	0.8 (−0.2, 1.7) *p = 0.138*	−1.6 (− 2.9, − 0.3) *p = 0.016*

Notes: 95% CI (confidence interval)

^a^ Fixed effect model with a lagged variable as the outcome, controlling for time-invariant confounders but constraining sex and age effects on the outcome to be fixed across time, and sample selection correction with the predictors of age, weekly spending money, school connectedness, and province.

^b^ based on adjusted estimates.

In the cross-sectional sample in 2020, 11.7% (*n* = 882) of respondents reported past year cannabis use. Among respondents who reported cannabis use in the past year, 14.1% (*n* = 124) reported solitary cannabis use one or two times in the past 30 days and 21.4% (*n* = 189) reported solitary cannabis use 3 or more time in the past 30 days. Overall, 27.3% (*n* = 241) of respondents who reported past year cannabis use reported that their cannabis use has increased because of COVID-19, whereas 23.2% (*n* = 205) reported their cannabis use had decreased because of COVID-19. Similarly, 27.4% (*n* = 242) of respondents reporting past year cannabis use also reported that they were using cannabis to cope with changes related to COVID-19.

## Discussion

This paper provides new insight on youth cannabis use. First, as it relates to the initial period of the COVID-19 pandemic, but more importantly by being among the first to examine the potential effect of the early COVID-19 pandemic period on cannabis use among youth in a prospective cohort with pre-pandemic data. Using survey data linked across three study waves, and adjusting for age of entry into the cohort and self-selection bias, there was no evidence of an adverse effect of the early stages of the COVID-19 pandemic period on cannabis use among a sample of youth in Ontario and Quebec, Canada. In fact, despite evidence of a general increase in cannabis use among youth in Canada prior to legalization among adults [29], it appears that cannabis use was potentially attenuated during the initial pandemic period (May to July 2020) as changes in cannabis use evident here were less than would have typically been expected over this time period [30]. Continuing prospective research is needed to examine the ongoing pandemic context on youth cannabis use.

Evidence from an online survey of Canadian adults suggests rates of cannabis use remained stable during the early pandemic period (May to June 2020) [31]. This is consistent with evidence from an online survey in Belgium during the early pandemic period (April 2020) that also identified that there were no significant changes in the consumption of cannabis use among adults due to COVID-19 restrictions [32]. However, we cannot assume the same among youth since substance use tends to escalate during adolescence as patterns of use become more established [4, 6]. Yet, it has also been suggested that since drug use among youth often happens in the context of peers [33], there may be a different response among youth given lockdown and social distancing restrictions associated with the COVID-19 pandemic [34]. This notion of a differential impact among youth is supported with a recent study from a small cross-sectional online sample of youth aged 16 to 18 years in Ontario (Canada) suggesting that the prevalence of cannabis use decreased 3 weeks after social distancing measures came into effect [15]. Similarly, our prospective evidence that the expected increase in cannabis use typically seen over time as youth progress through high school was attenuated during the early stages of the pandemic response further support it. We also identified that the initial pandemic period appears to have hosted a slowing in the progression of less frequent use (monthly) relative to more frequent use (weekly or daily). Considering evidence from youth that few regular users spontaneously reduce their use, and that among those who do, frequent users are predominant [5], the overall consistency across the three cannabis use frequencies adds legitimacy to our findings.

Interestingly, although research suggests males are more likely to escalate cannabis use relative to females during high school [4], we identified that during the early stages of the COVID-19 pandemic period, females appeared more apt to maintain (or escalate) use relative to males across all cannabis use outcomes modelled. While it cannot be determined with these data, it is possible that cannabis use among males may be more socially driven than among females [33, 34]. Given evidence that youth who reduce their cannabis are more likely to binge drink [6], and cannabis use tends to co-occur with alcohol and vaping among youth [6, 35], it is also possible that male youth may have transitioned between substances during the initial pandemic period. Moving forward, there is a need to both explore the pathways surrounding the sex differences identified here, and to use a similar modelling approach for examining additional substance use outcomes, specifically alcohol use, vaping (e-cigarettes), tobacco use, and poly-substance use.

In a small sample of U.S. adults, 12.5% reported using cannabis prior to the COVID-19 outbreak, whereas an additional 5% of respondents reported cannabis use onset at the start of the COVID-19 outbreak (April to May 2020) [36]. Similarly, in a sample of adults from Belgium during the early pandemic period, only 2.1% or cannabis users reported having used more cannabis that prior to the COVID-19 lockdown [32]. Our results suggest there is potentially a different phenomenon among youth, as substantially more youth in our sample who were existing cannabis users (27%), reported that their cannabis use had increased due to COVID-19 and that they were using cannabis to cope with COVID-19. This is consistent with the CCSA suggestion that conditions surrounding COVID-19 (e.g., stress and anxiety, social isolation due to physical distancing, closing of non-essential facilities) are likely to lead to increased cannabis use [13]. Further prospective studies are needed to disentangle the downstream impact of COVID-19 on cannabis use as a coping mechanism among youth.

### Strengths and limitations

Key strengths of this study include the prospective cohort design with a relatively large sample size, and the availability of early pandemic response data linked to 2 years of pre-COVID-19 data from youth in two Canadian provinces, allowing examination of within-individual effects and adjustment for age-related changes. While the COMPASS study is based on self-reported data, which can be prone to recall and social desirability bias, it uses passive consent protocols which is essential in self-report research for producing robust results that limit self-selection and response bias, particularly for measures of substance use behaviours [37, 38]; student names are not required, helping to preserve perceptions of anonymity for honest reporting. In terms of the design and statistical models, we built counterfactuals in the absence of a possible comparison group, but double difference models are limited by assuming parallel trends; therefore, there is no control for within-individual variations over time related to time-varying unobserved characteristics (e.g. changes in socioeconomic status). Possible limitations include the change from school-based paper-and-pencil questionnaires to online assessment, which may have influenced reports. The lower online response rates may bias the results; students not participating in the online survey may be at higher risk of cannabis use and its adverse impacts. Based on the previous in-school data collections, we utilized correction methods to mitigate the impact of self-selection into the 2020 wave. However, the consistency of estimates may be affected if there are departures from the statistical assumptions of sample selection models (e.g. assuming error terms that are jointly normally distributed). Lastly, COMPASS is based on a convenience sample of participating schools, so results may not be generalizable to all Canadian youth.

## Conclusion

In this large prospective study of youth, including pre-pandemic data and data collected in the period immediately after the initial pandemic restrictions were implemented, we identified that there does not appear to be a detrimental effect of the early stages of the COVID-19 pandemic period on youth cannabis use, after adjusting for age-related changes. Further prospective research is needed to explore the impact of the ongoing pandemic context on youth cannabis use onset and progression.

## Data Availability

The datasets used and/or analysed during the current study available from the corresponding author on reasonable request submitted via the following online application form (https://uwaterloo.ca/compass-system/sites/ca.compass-system/files/uploads/files/compass_data_use_application_2020.pdf).

## Abbreviations

CCSA: Canadian Centre on Substance Use and Addiction
COMPASS: The Cannabis use, Obesity, Mental health, Physical activity, Alcohol use, Smoking, and Sedentary behaviour Study
GSEM: Generalized Structural Equation Modeling
SEM: Structural Equation Model
